# Exploration of the Combination of PLK1 Inhibition with Immunotherapy in Cancer Treatment

**DOI:** 10.1155/2018/3979527

**Published:** 2018-12-02

**Authors:** Mengyuan Li, Zhixian Liu, Xiaosheng Wang

**Affiliations:** ^1^Biomedical Informatics Research Lab, School of Basic Medicine and Clinical Pharmacy, China Pharmaceutical University, Nanjing 211198, China; ^2^Cancer Genomics Research Center, School of Basic Medicine and Clinical Pharmacy, China Pharmaceutical University, Nanjing 211198, China; ^3^Big Data Research Institute, China Pharmaceutical University, Nanjing 211198, China

## Abstract

**Background:**

PLK1 overexpression is oncogenic and is associated with poor prognosis in various cancers. However, the current PLK1 inhibitors have achieved limited clinical successes. On the other hand, although immunotherapies are demonstrating efficacy in treating many refractory cancers, a substantial number of patients do not respond to such therapies. The potential of combining PLK1 inhibition with immunotherapy for cancer treatment is worthy of exploration.

**Methods:**

We analyzed the associations of* PLK1* expression with tumor immunity in 33 different cancer types. Moreover, we analyzed the associations of the drug sensitivities of PLK1 inhibitors with tumor immunity in cancer cell lines. Furthermore, we explored the mechanism underlying the significant associations between PLK1 and tumor immunity. Finally, we experimentally verified some findings from bioinformatics analysis.

**Results:**

The cancers with higher* PLK1* expression levels tended to have lower immune activities, such as lower HLA expression and decreased B cells, NK cells and tumor-infiltrating lymphocytes infiltration. On the other side, elevated tumor immunity likely increased the sensitivity of cancer cells to PLK1 inhibitors. The main mechanism underlying the associations between PLK1 and tumor immunity may lie in the aberrant cell cycle and p53 pathways in cancers.

**Conclusions:**

Our findings implicate that the PLK1 inhibition and immunotherapy combination may achieve a synergistic antitumor efficacy.

## 1. Introduction

PLK1 (Polo-like kinase 1) is a member of the Polo-like kinase family [1], which plays an important role in cell cycle regulation [2]. The role PLK1 plays in regulating cell cycle is diverse which includes controlling mitotic entry, harmonizing centrosome and cell cycles, regulating chromosome segregation, and mediating cytokinesis and meiosis [2]. Thus, the malfunction of PLK1 would result in cell cycle aberration that often incites cell proliferation. In fact, a substantial number of studies have revealed that PLK1 was overexpressed in a wide variety of cancers, and its overexpression correlated with unfavorable prognosis of cancer patients [3]. Hence, the inhibition of PLK1 has been suggested as a potential strategy for cancer therapy [4]. A number of PLK1 inhibitors have been explored in laboratory or clinical studies such as BI2536, volasertib, GSK461364, rigosertib, poloxin, poloxin-2, and RO3280 [3]. However, none of these exploratory agents have been used in clinics thus far [5].

On the other hand, recently cancer immunotherapy is demonstrating astonishing successes in treating various cancers [6, 7]. Particularly, the blockade of immune checkpoints CTLA4 (cytotoxic T-lymphocyte-associated protein 4), PD1 (programmed cell death protein 1), and PD-L1 (programmed cell death 1 ligand) has clinical successes in various cancers including melanoma, lung cancer, renal cell cancer, bladder cancer, head and neck cancer, Hodgkin's lymphoma, and the cancers with MSI (microsatellite instability) or DNA mismatch-repair deficiency [6]. Another notable cancer immunotherapeutic strategy is the chimeric antigen receptor (CAR) T cell therapy that has been used to treat refractory leukemia and lymphoma successfully [7]. Despite these remarkable achievements of cancer immunotherapy, a substantial proportion of patients had limited or no response to such therapies [8]. To predict the patients responsive to cancer immunotherapy, some biomarkers have been explored such as tumor mutation burden (TMB) [9, 10], neoantigens [11], MSI [12], and PD-L1 expression [13]. In addition, to improve the efficacy of cancer immunotherapy, the combination of immunotherapy with chemotherapy, radiotherapy, or targeted therapies has been explored [14]. For example, a recent study demonstrated that the combination of cyclin-dependent kinases 4 and 6 (CDK4/6) inhibitors with immunotherapy could promote antitumor immunity [15].

In this study, to explore the potential of combining PLK1 inhibitors with immunotherapy in treating cancers, we analyzed the associations of* PLK1* expression with immune cell infiltration and immune activities in 33 different cancer types based on the Cancer Genome Atlas (TCGA) data (https://cancergenome.nih.gov/). Moreover, we analyzed the associations of the drug sensitivities of PLK1 inhibitors with immune cell infiltration and immune activities in cancer cell lines (CCLs) based on the Genomics of Drug Sensitivity in Cancer (GDSC) project data (http://www.cancerrxgene.org/). Furthermore, we explored the potential mechanisms that underlie the significant associations between* PLK1* expression and tumor immunity.

## 2. Methods

### 2.1. Datasets

The TCGA data for gene expression profiles (RNA-Seq, Level 3) and gene somatic mutations (Level 3) were downloaded from the genomic data commons data portal (https://portal.gdc.cancer.gov/). The 33 TCGA cancer types analyzed are shown in Table 1. The GDSC data for gene expression profiles (Affymetrix Human Genome U219 array) and drug sensitivities (IC50) were downloaded from the Wellcome Sanger Institute website: https://www.cancerrxgene.org/downloads. We analyzed the enrichment levels of 6 immune cell types and functions in cancers including B cells, natural killer (NK) cells, tumor-infiltrating lymphocytes (TILs), human leukocyte antigen (HLA), regulatory T (Treg) cells, and cancer-testis antigens (CTAs) based on the expression profiles of their gene signatures. These gene signatures are shown in the Supplementary Table S1.

### 2.2. Evaluation of the Activity of an Immune Cell Type or Function in Cancers

We quantified the activity (or enrichment levels) of an immune cell type or function in a cancer sample using the single-sample gene-set enrichment analysis (ssGSEA) score [16, 17]. The gene-set is the set of gene signatures of the immune cell type or function. The higher the ssGSEA score, the higher the activity of the immune cell type or function. In addition, we assessed the levels of immune infiltration in cancers by the ESTIMATE algorithm [18]. ESTIMATE output immune scores quantify the immune infiltration levels in cancers based on gene expression profiles data.

### 2.3. Cell Lines and Cell Culture

Human cells from Lung Squamous Cell Carcinoma (SK-MES-1), Glioblastoma Multiforme (U251), Uterine Corpus Endometrial Carcinoma (HEC-1B), and Skin Cutaneous Melanoma (SK-MEL-2) were from the American Type Culture Collection (ATCC). SK-MES-1 and U251 cells were cultured in DMEM (GIBCO, USA) supplemented with 10% fetal bovine serum (FBS, GIBCO, USA). HEC-1B cells were incubated in Roswell Park Memorial Institute-1640 (RPMI-1640, GIBCO, USA) supplemented with 10% FBS. SK-MEL-2 was cultured in MEM (GIBCO, USA) supplemented with 10% FBS. All the cells were cultured in a humidified incubator at 37°C and a 5% CO_2_ atmosphere. Cells were harvested in logarithmic growth phase in all the experiments performed in this study.

### 2.4. Reverse Transcription Quantitative PCR (qPCR) Analysis

BI2536 were purchased from Selleck. Cells were harvested after being treated with BI2536 (1 *μ*M, 48h). The total RNA was isolated by Trizol (Invitrogen, USA) and was reversely transcribed into cDNA by the RevertAid First Strand cDNA Synthesis Kit (Thermo Fisher, USA). Primer sequences used for qPCR were presented in Supplementary Table S2. Primers were diluted in nuclease-free water with the real-time PCR (RT-PCR) Master Mix (SYBR Green) (TOYOBO Co., LTD, JAPAN). Relative copy number was determined by calculating the fold-change difference in the gene of interest relative to *β*-actin. The qPCR was performed on an ABI 7500 FAST and Applied Biosystems StepOnePlus RT-PCR machine.

### 2.5. Flow Cytometry

Cells were harvested after treatments for 48 hours by trypsinization and were washed with PBS. Cells were resuspended in labeling buffer (PBS supplemented with 10% FBS and 1% NaN3) to a final concentration of 5×105 per ml and were stained with W6/32 monoclonal antibody (1:20,eBIOSCIENCE: 12-9983-42) at 37°C without lights for 30 minutes. Cells were then washed by PBS for flow cytometric analysis using a LSRII 4-laser flow cytometer (Becton Dickinson, USA). The results were analyzed and MFI calculated by FlowJo.

### 2.6. Statistical Analyses

We calculated the correlation between the* PLK1* expression levels and the expression levels of another gene using the Pearson method, and the correlations between the* PLK1* expression levels and the other variables including the enrichment levels (ssGSEA scores) of a gene-set, tumor mutation counts, and drug sensitivities (IC50) using the Spearman method. In comparisons of* TP53* mutation rates between the cancers with higher* PLK1* expression levels (upper third) and the cancers with lower* PLK1* expression levels (lower third), we used the Fisher's exact test. We adjusted for multiple tests using the false discovery rate (FDR) calculated by the Benjamini and Hochberg (BH) method [19]. The threshold of FDR < 0.1 was used to identify the statistical significance. All the computational and statistical analyses were implemented by R (https://www.r-project.org/). The experimental data were analyzed by Prism 5.0 software (GraphPad) and were presented as mean ± SD. The* t* test P < 0.05 was considered statistically significant.

## 3. Results

### 3.1. *PLK1* Expression Likely Correlates with Depressed Immune Cell Infiltration and Immune Activities in Cancer

**Figure 1:**
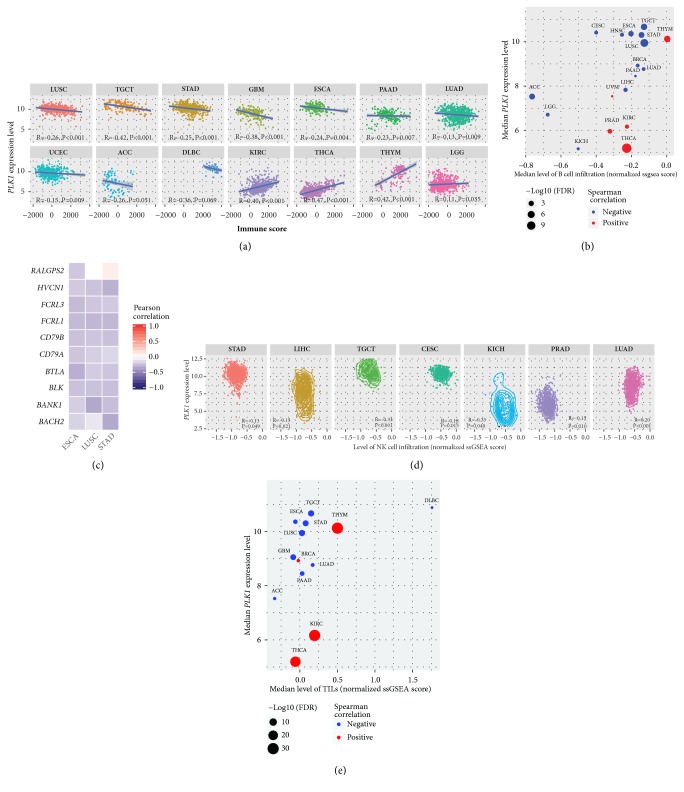
**PLK1 likely inversely correlates with immune cell infiltration and immune activities in cancer.** (a) The* PLK1* expression levels inversely correlate with immune scores in 10 cancer types and positively correlate with immune scores in 4 cancer types (Spearman correlation, FDR<0.1). (b) The* PLK1* expression levels inversely correlate with B cell infiltration in 13 cancer types and positively correlate with B cell infiltration in 5 cancer types (Spearman correlation, FDR<0.1). (c) Most of the B cell gene signatures show negative expression correlations with the* PLK1* expression in ESCA, LUSC, and STAD (Pearson correlation, FDR<0.1). (d) The* PLK1* expression levels inversely correlate with NK cell infiltration in 6 cancer types and positively correlate with NK cell infiltration in 1 cancer type (Spearman correlation, FDR<0.1). (e) The* PLK1* expression levels inversely correlate with TILs infiltration in 9 cancer types and positively correlate with TILs infiltration in 4 cancer types (Spearman correlation, FDR<0.1). R: Spearman or Pearson correlation coefficient. FDR: false discovery rate. NK: natural killer. TILs: tumor-infiltrating lymphocytes.

We found that the* PLK1* expression levels were negatively associated with immune scores in 10 cancer types (LUSC, TGCT, STAD, GBM, ESCA, PAAD, LUAD, UCEC, ACC, and DLBC) while were positively associated with immune scores in 4 cancer types (KIRC, THCA, THYM, and LGG) (Spearman correlation, FDR<0.1) (Figure 1(a)) among the 33 cancer types analyzed. Moreover, higher* PLK1* expression levels were associated with more abundant B cell infiltration in 13 cancer types (LUSC, TGCT, ACC, STAD, ESCA, LIHC, HNSC, LGG, CESC, BRCA, LUAD, KICH, and PAAD) while were associated with fewer B cell infiltration in 5 cancer types (THCA, THYM, PRAD, KIRC, and UVM) (Spearman correlation, FDR<0.1) (Figure 1(b)). Notably, all the 10 B cell gene signatures (*BANK1*,* HVCN1*,* CD79B*,* RALGPS2*,* FCRL3*,* CD79A*,* BACH2*,* FCRL1*,* BLK,* and* BTLA*) showed negative expression correlations with the* PLK1* expression in ESCA, and 9 did in LUSC and STAD (Pearson correlation, FDR<0.1) (Figure 1(c)). In addition, in 6 cancer types (TGCT, PRAD, CESC, LIHC, KICH, and STAD), the upregulation of* PLK1* was associated with higher levels of NK cell infiltration, and in 1 cancer type (LUAD), we observed an opposite trend (Spearman correlation, FDR<0.1) (Figure 1(d)). Furthermore, we associated the* PLK1* expression levels with the enrichment levels of TILs in cancers. We found that the* PLK1* expression levels negatively correlated with the enrichment levels of TILs in 9 cancer types (LUSC, TGCT, STAD, GBM, PAAD, ESCA, LUAD, ACC, and DLBC), and in 4 cancer types (KIRC, THYM, THCA, and BRCA), we observed an opposite trend (Spearman correlation, FDR<0.1) (Figure 1(e)). Notably, 114 (95%) of the 120 TILs genes showed negative expression correlations with the* PLK1* expression in LUSC, and 110 (92%) TILs genes did in TGCT (Spearman correlation, FDR<0.1). Altogether, these data suggest that elevated* PLK1* expression tends to inhibit immune cell infiltration and antitumor immunity in a number of cancer types.

### 3.2. *PLK1* Expression Likely Correlates with Depressed HLA Activity in Cancer

**Figure 2:**
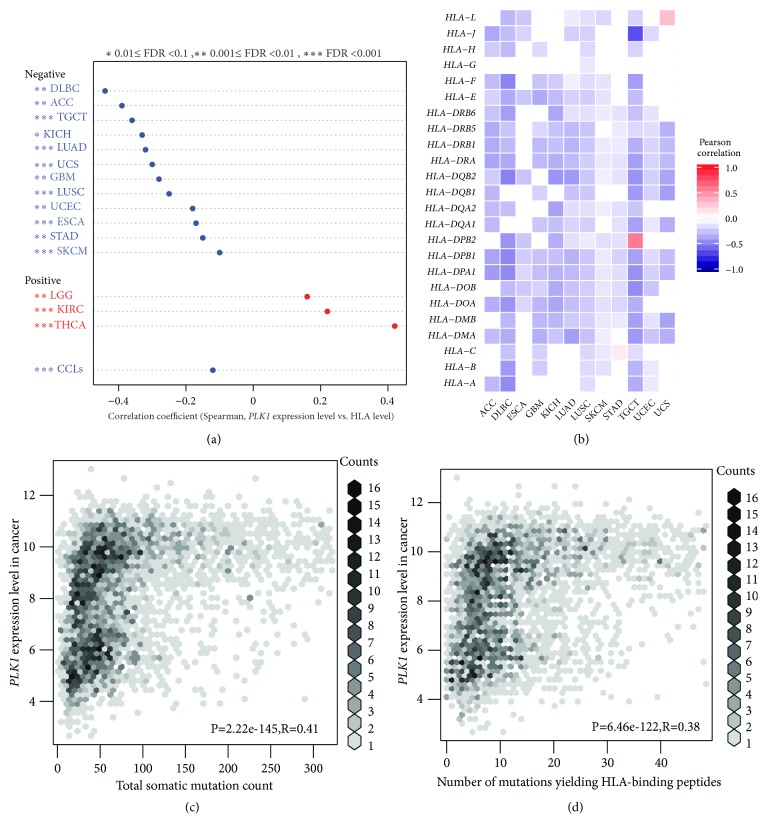
**PLK1 likely inversely correlates with HLA activity in cancer.** (a) The* PLK1* expression levels inversely correlate with the HLA activity in 12 cancer types and cancer cell lines and positively correlate with the HLA activity in 3 cancer types (Spearman correlation, FDR<0.1). (b) Most of the 24 HLA genes show negative expression correlations with the* PLK1* expression in the 12 cancer types in which the* PLK1* expression inversely correlates with the HLA activity (Pearson correlation, FDR<0.1). (c) The* PLK1* expression levels positively correlate with somatic mutation counts in cancer. (d) The* PLK1* expression levels positively correlate with the numbers of mutations yielding predicted HLA-binding peptides. HLA: human leukocyte antigen. CCLs: cancer cell lines. R: Spearman or Pearson correlation coefficient. FDR: false discovery rate.

HLA genes encode the MHC (major histocompatibility complex) proteins that are important in the tumor immune regulation [20]. We found that the* PLK1* expression negatively correlated with the HLA activity in 12 cancer types (LUAD, LUSC, TGCT, GBM, ACC, UCEC, DLBC, STAD, KICH, ESCA, SKCM, and UCS), while in 3 cancer types (THCA, KIRC, and LGG), we observed an opposite trend (Spearman correlation, FDR<0.1) (Figure 2(a)). The GDSC data analysis showed that the* PLK1* expression negatively correlated with the HLA activity in cancer cell lines (Spearman correlation, R=-0.12, P=2.0*∗*10^−4^) (Figure 2(a)). Furthermore, we found that a majority of the 24 HLA genes analyzed showed significantly negative expression correlations with the* PLK1* expression in the 12 cancer types in which the* PLK1* expression negatively correlated with the HLA activity. For example, in both lung cancer types, all the 24 HLA genes had negative expression correlations with the* PLK1* expression in LUSC, and 19 HLA genes did in LUAD (Pearson correlation, FDR<0.1) (Figure 2(b)). 22 and 21 HLA genes showed negative expression correlations with the* PLK1* expression in TGCT and DLBC, respectively. Notably,* HLA-A*,* HLA-DPA1*,* HLA-DQB1*,* HLA-DRA*,* HLA-DRB1*,* HLA-DRB5,* and* HLA-J* consistently negatively correlated with the* PLK1* expression in the 12 cancer types (Figure 2(b)). Taken together, these results suggest that the* PLK1* expression likely inhibits the HLA activity in cancer.

The neoantigens yielded by gene mutations are associated with antitumor immunity [11]. We found that the tumors with higher* PLK1* expression levels had significantly higher total somatic mutation counts than the tumors with lower* PLK1* expression levels in TCGA (Spearman correlation, R=0.46, P=2.57*∗*10^−214^) (Figure 2(c)). Moreover, the tumors more highly expressing* PLK1* had significantly more mutations yielding predicted HLA-binding peptides [21] than the tumors more lowly expressing* PLK1* (Spearman correlation, R=0.43, P=1.03*∗*10^−186^) (Figure 2(d)). It suggests that although the* PLK1* upregulation correlates with higher TMB and more neoantigens, it inhibits antitumor immune response by repressing the HLA activity.

### 3.3. *PLK1* Expression Likely Correlates with Depressed Regulatory T Cell Activity in Cancer

**Figure 3:**
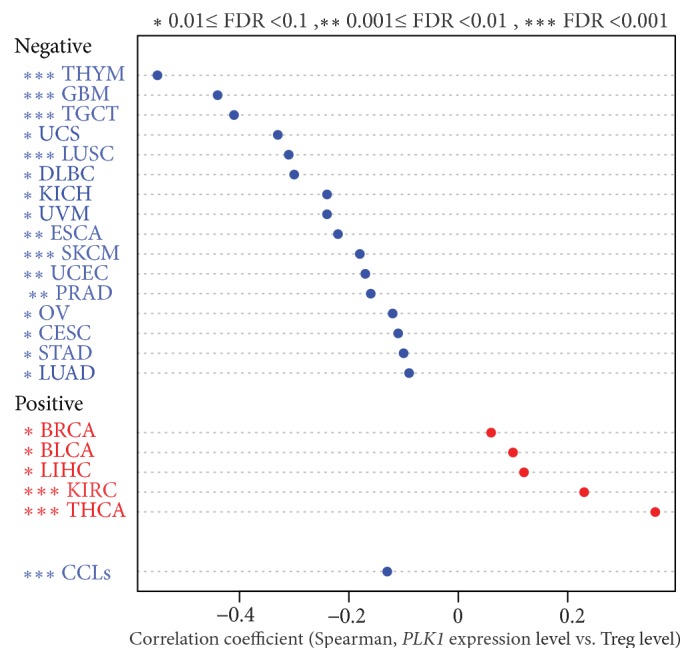
**PLK1 likely inversely correlates with regulatory T cell activity in cancer.** Treg: regulatory T. CCLs: cancer cell lines.

Treg cells play an important role in tumor immunosuppression [22]. We found that high* PLK1* expression levels were associated with depressed Treg cell enrichment levels in 16 cancer types (THYM, LUSC, GBM, TGCT, SKCM, PRAD, UCEC, ESCA, UCS, UVM, OV, DLBC, LUAD, STAD, CESC, and KICH) while were associated with enhanced Treg cell activity in 5 cancer types (THCA, KIRC, LIHC, BRCA, and BLCA) (Spearman correlation, FDR<0.1) (Figure 3). The GDSC data analysis showed that high* PLK1* expression levels were associated with decreased Treg cell enrichment levels in cancer cell lines (Spearman correlation, R=-0.13, P=3.29*∗*10^−5^) (Figure 3). Altogether, these data suggest that the* PLK1* expression is negatively associated with the Treg cell activity in a wide range of cancers.

### 3.4. *PLK1* Expression Likely Positively Correlates with Expression of Cancer-Testis Antigens in Cancer

**Figure 4:**
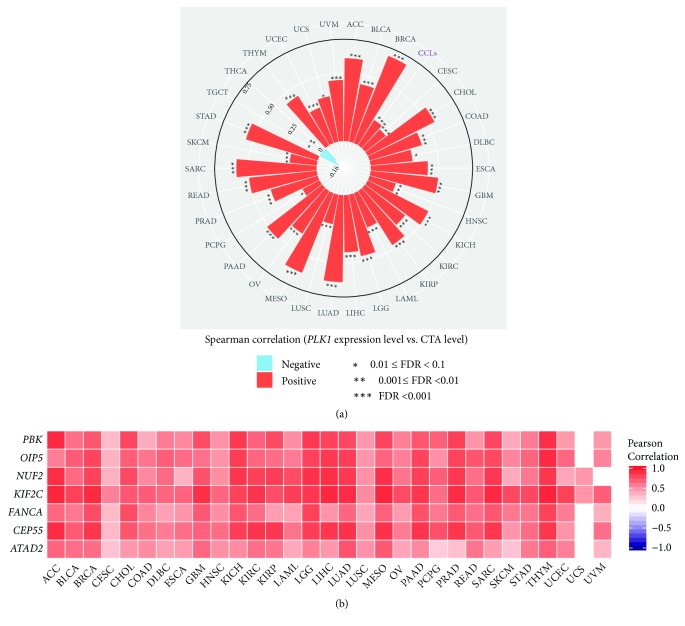
**PLK1 likely positively correlates with cancer-testis antigen (CTA) activity in cancer.** (a) The* PLK1* expression levels are positively associated with the CTA enrichment levels in 31 cancer types (Spearman correlation, FDR<0.1). The Spearman correlation coefficient for each cancer type is proportional to the length of the bar pointing to the cancer type. (b) The CTA genes* ATAD2*,* CEP55*,* FANCA*,* KIF2C*,* NUF2*,* OIP5*, and* PBK* show significantly positive expression correlations with the* PLK1* expression in 30 cancer types (Pearson correlation, FDR<0.1).

CTAs are the immunogenic proteins that are aberrantly activated in many cancers [23]. Strikingly, we found that higher* PLK1* expression levels were significantly associated with higher CTA enrichment levels in 31 of the 33 cancer types (Spearman correlation, FDR<0.1) (Figure 4(a)). Markedly, the CTA genes* ATAD2*,* CEP55*,* FANCA*,* KIF2C*,* NUF2*,* OIP5*, and* PBK *had significantly positive expression correlations with the* PLK1* expression in 30 cancer types (Pearson correlation, FDR<0.1) (Figure 4(b)). Moreover, 166 (74%) of the 223 CTA genes showed positive expression correlations with the* PLK1* expression in LIHC, and 145 (65%) CTA genes did in KIRC. Furthermore, the GDSC data analysis showed that the* PLK1* expression levels were positively associated with the CTA enrichment levels in cancer cell lines (Spearman correlation, R=0.22, P=4.09*∗*10^−12^) (Figure 4(a)). Altogether, these data suggest that higher* PLK1* expression is associated with higher CTA presentation.

### 3.5. Elevated Immune Activities Tends to Enhance the Sensitivity of Cancer Cells to PLK1 Inhibitors

**Figure 5:**
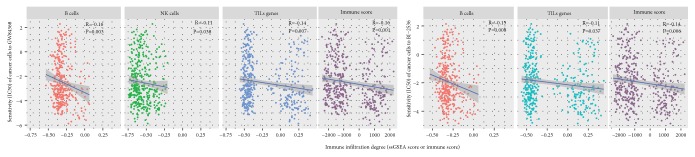
**Cancer immune activities positively correlate the sensitivity of cancer cells to PLK1 inhibitors (GW84368 and BI-2536).** ssGSEA: the single-sample gene-set enrichment analysis.

The GDSC data involved the drug sensitivity (IC50) of cancer cells to hundreds of compounds, of which GW84368 and BI-2536 target PLK1. We found that the enrichment levels of B cells, NK cells, and TILs negatively correlated with the IC50 values of GW84368, and the enrichment levels of B cells and TILs negatively correlated with the IC50 values of BI-2536 (Spearman correlation, P<0.05) (Figure 5). It indicated that higher levels of B cells, NK cells, or TILs could promote the sensitivity of cancer cells to PLK1 inhibitors. It is rational in that higher levels of B cells, NK cells, or TILs are associated with lower levels of PLK1 that would need lower concentrations of PLK1 inhibitors to inhibit cancer cell proliferation. Furthermore, the GDSC data analysis showed that the IC50 values of both GW84368 and BI-2536 were negatively associated with the immune scores of cancer cells (Spearman correlation, P<0.01) (Figure 5), again suggesting that elevated immune activities increase the sensitivity of cancer cells to PLK1 inhibitors.

### 3.6. PLK1 Inhibits Antitumor Immunity* via* the Cell Cycle Regulation

**Figure 6:**
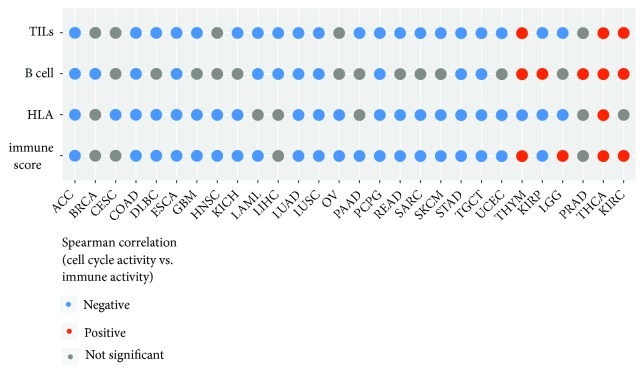
Immune activities likely inversely correlate with cell cycle activity in cancer.

PLK1 is one of the essential regulators of cell cycle progression [24]. As expected, the TCGA data analysis showed that the* PLK1* expression levels strongly correlated with the cell cycle activity in a positive direction in all 33 cancer types (Supplementary Table S3). Furthermore, our analysis showed that the high cell cycle activity tended to inhibit antitumor immunity. For example, the cell cycle activity negatively correlated with the TILs enrichment in 20 cancer types versus in 3 cancer types positively correlating with the TILs enrichment (Spearman correlation, FDR<0.1) (Figure 6). The cell cycle activity negatively correlated with the HLA enrichment in 21 cancer types, while only in 1 cancer type showed a positive correlation (Figure 6). Moreover, the cell cycle activity negatively correlated the B cell enrichment in 11 cancer types versus in 5 cancer types positively correlating with the B cell enrichment (Figure 6). Interestingly, the cell cycle showed a positive correlation with the CTA enrichment in 30 of the 33 cancer types (Spearman correlation, FDR<0.1) (Supplementary Table S4). Furthermore, we found that in 20 cancer types the cell cycle activity was negatively associated with the immune score compared to in 4 cancer types the cell cycle activity being positively associated with the immune score (Figure 6). Altogether, these results suggest that the* PLK1* upregulation inhibits antitumor immunity* via* enhancing the cell cycle activity in cancer. This is in line with a recent study showing that the cell cycle inhibition promoted antitumor immunity [15].

### 3.7. PLK1 Inhibition Increases MHC Class I Expression in Multiple Cell Lines

**Figure 7:**
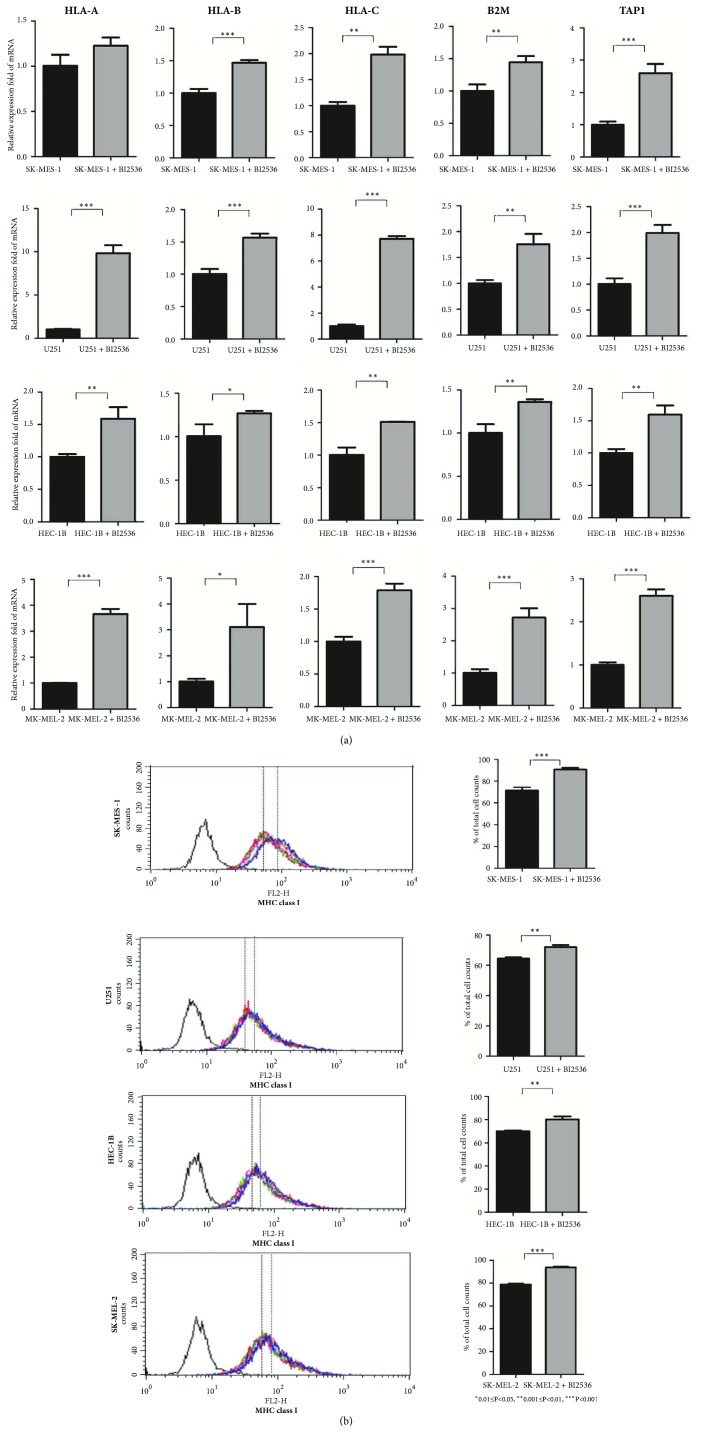
**PLK1 inhibition increases MHC class I expression in cell lines.** (a) qPCR analysis of MHC class I mRNA expression in pre- and post- treated cell lines with BI2536. (b) Flow cytometric analysis of MHC class I (HLA-ABC, W6/32 labeling) expression on pre- and post- treated cell lines with BI2536.

We used the PLK1 inhibitor (BI2536) to treat cancer cell lines and compared expression levels of MHC class I (*HLA-A*,* HLA-B*,* HLA-C*,* B2M,* and* TAP1* genes and their protein products) between the pre- and post- treated cell lines. We observed that the MHC class I molecules had significantly increased expression in the post-treated cell lines compared to the pretreated cell lines, and the results were consistent in all the four different cell lines (LUSC, GBM, UCEC, and SKCM) tested (Figure 7). This experiment verified the results from bioinformatics analysis that the* PLK1* expression inversely correlated with the HLA activity in diverse cancer types.

## 4. Discussion

**Figure 8:**
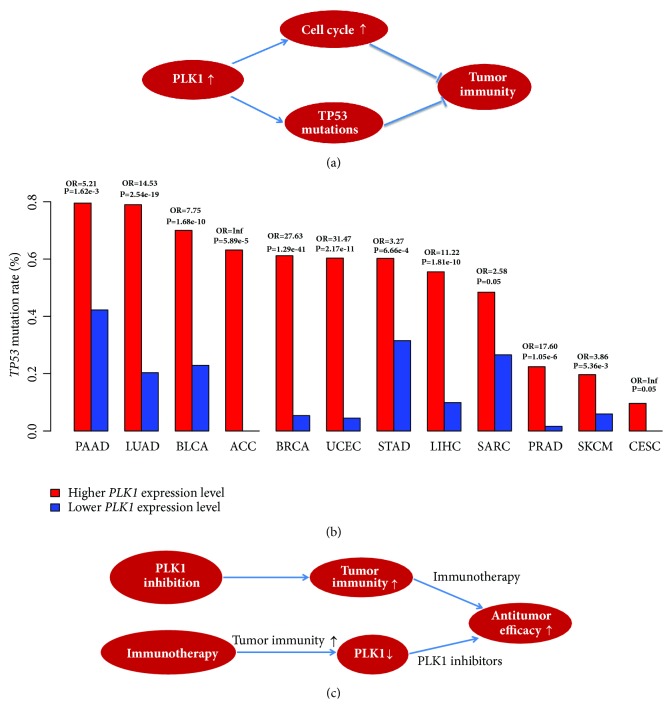
**The mechanism of PLK1 inhibiting tumor immunity and the viability of the PLK1 inhibition and immunotherapy combination in cancer treatment.** (a) PLK1 inhibits tumor immunity* via* regulating the cell cycle and p53 pathways. (b) The cancers with higher PLK1 expression levels show a higher prevalence of* TP53* mutations compared to the cancers with lower* PLK1* expression levels in 12 cancer types (Fisher's exact test, FDR<0.05). OR: odds ratio. (c) The PLK1 inhibition and immunotherapy combination is viable in promoting the antitumor efficacy.

PLK1 is a master regulator of cell cycle, and its overexpression is oncogenic in various cancer types. Thus, targeting PLK1 could be promising in treating a wide range of malignancies. However, the current PLK1 inhibitors have achieved very limited clinical successes. On the other hand, although immunotherapies are achieving rapid clinical successes in treating many refractory cancers, a considerable number of patients do not respond to such therapies. To improve the clinical efficacy of both therapies, the combination of PLK1 inhibition and immunotherapy merits consideration. To explore the possibility of combining both therapies, we analyzed the associations between* PLK1* expression and tumor immunity in various different cancer types. Our bioinformatics analyses showed that* PLK1* expression tended to inhibit antitumor immunity as the cancers with higher* PLK1* expression levels often had lower HLA expression levels and TILs infiltration. Moreover, the in vitro experiment verified that the PLK1 inhibition significantly increased the expression of HLA molecules in various cancers. A main mechanism by which PLK1 inhibits antitumor immunity lies in that the PLK1 upregulation activates the cell cycle which may decrease tumor immunogenicity (Figure 8(a)). Besides, we found that the cancers with higher* PLK1* expression levels had significantly higher frequency of* TP53* mutations than the cancers with lower* PLK1* expression levels in 12 cancer types (Fisher's exact test, FDR<0.05) (Figure 8(b)), suggesting that the* PLK1 *upregulation positively correlates with the prevalence of* TP53* mutations. Hence, the higher* TP53* mutation rates in the cancers with higher* PLK1* expression levels may also be partly responsible for the depressed tumor immunity in these cancers (Figure 8(a)) since a prior study has demonstrated that wildtype p53 could promote tumor immunity [25].

The correlations of* PLK1* expression with the immune signature could be affected by other factors such as patient age, gender, tumor stage, and grade. We re-analyzed the correlations of* PLK1* expression with the immune signature (immune score) under the stratification of patients based on age (<60 and ≥60 years old), gender (male and female), stage (early stage (Stage I-II) and late stage (Stage III-IV)), and grade (low-grade (G1-2) and high-grade (G3-4)), respectively. We did not observe marked changes of the statistical correlations when these covariates were considered (Supplementary Table S5). In addition, we performed the multiple linear regression analysis of the correlations between* PLK1* expression and the immune signature by adding the covariate “age”. Our results showed that the correlations between* PLK1* expression and the immune signature were unlikely affected by the variable “age” (Supplementary Table S6).

It is rational to anticipate that the PLK1 inhibition and immunotherapy combination may improve the antitumor efficacy. First, PLK1 inhibition is capable of boosting tumor antigen presentation and antitumor immune infiltration, which can be further augmented by the addition of immunotherapy (Figure 8(c)). Second, cancer immunotherapy may enhance tumor immunogenicity, which in turn increases the sensitivity of cancer cells to PLK1 inhibitors (Figure 8(c)). Hence, the PLK1 inhibition and immunotherapy combination could be promising in cancer treatment, although it needs to be proved by further experimental and clinical validations.

## 5. Conclusions

PLK1 likely inhibits antitumor immunity, and the elevated tumor immunity may enhance the sensitivity of cancer cells to PLK1 inhibitors. It implicates that the combination of PLK1 inhibition and immunotherapy may achieve a synergistic antitumor efficacy.

## Figures and Tables

**Table 1:** The 33 TCGA cancer types analyzed.

**Cancer type**	**Full name**	**# Cancer samples**
ACC	adrenocortical carcinoma	79

BLCA	bladder urothelial carcinoma	408

BRCA	breast invasive carcinoma	1100

CESC	cervical squamous-cell carcinoma and endocervical adeno-carcinoma	306

CHOL	cholangiocarcinoma	36

COAD	colon adenocarcinoma	287

DLBC	lymphoid neoplasm diffuse large B-cell lymphoma	48

ESCA	esophageal carcinoma	185

GBM	glioblastoma multiforme	166

HNSC	head and neck squamous cell carcinoma	522

KICH	kidney chromophobe	66

KIRC	kidney renal clear cell carcinoma	534

KIRP	kidney renal papillary cell carcinoma	291

LAML	acute myeloid leukemia	173

LGG	brain lower-grade glioma	530

LIHC	liver hepatocellular carcinoma	373

LUAD	lung adenocarcinoma	517

LUSC	lung squamous cell carcinoma	501

MESO	mesothelioma	87

OV	ovarian serous cystadenocarcinoma	307

PAAD	pancreatic adeno-carcinoma	179

PCPG	pheochromocytoma and paraganglioma	184

PRAD	prostate adenocarcinoma	498

READ	rectum adenocarcinoma	95

SARC	sarcoma	263

SKCM	skin cutaneous melanoma	472

STAD	stomach adenocarcinoma	415

TGCT	testicular germ-cell tumors	156

THCA	thyroid carcinoma	509

THYM	thymoma	120

UCEC	uterine corpus endometrial carcinoma	370

UCS	uterine carcinosarcoma	57

UVM	uveal melanoma	80

## Data Availability

The data for tumor tissue can be downloaded from the genomic data commons data portal (https://portal.gdc.cancer.gov/), and the data for cancer cell lines can be downloaded from the website: https://www.cancerrxgene.org/downloads.

## Abbreviations

PLK1: Polo-like kinase 1
CTLA4: Cytotoxic T-lymphocyte-associated protein 4
PD1: Programmed cell death protein 1
PD-L1: Programmed cell death 1 ligand
CDK4/6: Cyclin-dependent kinases 4 and 6
TILs: Tumor-infiltrating lymphocytes
HLA: Human leukocyte antigen
MHC: Major histocompatibility complex
Treg: Regulatory T
CTAs: Cancer-testis antigens
MSI: Microsatellite instability
CAR: Chimeric antigen receptor
TMB: Tumor mutation burden
FDR: False discovery rate
ssGSEA: Single-sample gene-set enrichment analysis
TCGA: The Cancer Genome Atlas
GDSC: Genomics of Drug Sensitivity in Cancer
CCLs: Cancer cell lines
qPCR: Quantitative PCR
RT-PCR: Real-time PCR.

## Supplementary Materials

Supplementary Materials Table S1: the gene signatures for the 6 immune cell types and functions analyzed. Table S2: primer sequences used for qPCR analysis. Table S3: correlations between the* PLK1* expression levels and the cell cycle activity in 33 TCGA cancer types. Table S4: correlations between the CTA enrichment levels and the cell cycle activity in 33 TCGA cancer types. Table S5: the correlations of* PLK1* expression with immune scores. Table S6: the multiple linear regression analysis of the correlations between* PLK1 *expression and the immune signature by adding the covariate “age”.
